# Household hunger trajectories and mental health symptoms of adolescent girls and young women in rural South Africa: an HPTN-068 longitudinal study

**DOI:** 10.64898/2026.09.01.26361988

**Published:** 2026-09-03

**Authors:** Rishika Chakraborty, Molly Rosenberg, M. Margaret Weigel, Audrey Pettifor, Kathleen Kahn, F. Xavier Gómez-Olivé

## Abstract

**Purpose:**

Despite the high documented prevalence of hunger and poor mental health in adolescent girls and young women (AGYW) in South Africa, this relationship remains understudied, with existing studies limited by their cross-sectional designs. This longitudinal study aimed to identify the association of hunger trajectories with anxiety and depressive symptoms, and hope in AGYW.

**Methods:**

We used secondary data from the HIV Prevention Trials Network (HPTN) -068 conducted in rural Agincourt, South Africa. Complete data from 1779 AGYW collected at baseline (2011/12) and three annual follow-up visits were used. Hunger trajectories, measured using the Household Hunger Scale, were estimated via Group-Based Trajectory Modelling. Self-reported incident anxiety and depressive symptoms and hope were assessed based on AGYW’s last two follow-up visits. Covariate adjusted modified Poisson regression models estimated the association between hunger trajectories and incident anxiety symptoms, incident depressive symptoms, and hope.

**Results:**

Moderate-severe hunger was prevalent in 11.0%, 10.8%, and 6.0% of the households at baseline, follow-up 1, and 2, respectively. Incident anxiety symptoms were reported by 4.5%, incident depressive symptoms by 20.0% and hopelessness by 52.8% of the AGYW. Two hunger trajectories were identified- no hunger (82%) and marginal hunger (18%). Hunger trajectories were not associated with incident anxiety symptoms [RR:1.09, 95% CI: 0.55, 2.18], incident depressive symptoms [RR: 0.97; 95% CI: 0.72, 1.33] nor hope [RR: 1.00; 95% CI: 0.81, 1.23] in AGYW.

**Conclusion:**

Better understanding of the factors that promote resiliency and mental health of AGYW in this setting is warranted to inform the design of interventions.

## Full Text Availability

The license terms selected by the author(s) for this preprint version do not permit archiving in PMC. The full text is available from the preprint server.

